# Multifunctional interaction of CihC/FbpC orthologs of relapsing fever spirochetes with host-derived proteins involved in adhesion, fibrinolysis, and complement evasion

**DOI:** 10.3389/fimmu.2024.1390468

**Published:** 2024-04-25

**Authors:** Ann-Sophie Damm, Flavia Reyer, Luisa Langhoff, Yi-Pin Lin, Franco Harald Falcone, Peter Kraiczy

**Affiliations:** ^1^ Institute of Medical Microbiology and Infection Control, University Hospital of Frankfurt, Goethe University Frankfurt, Frankfurt, Germany; ^2^ Department of Infectious Disease and Global Health, Cummings School of Veterinary Medicine, Tufts University, North Grafton, MA, United States; ^3^ Institute of Parasitology, Biomedical Research Center Seltersberg, Justus Liebig University Giessen, Giessen, Germany

**Keywords:** relapsing fever, spirochetes, *Borrelia*, complement, immune evasion, host cell interaction, fibronectin, plasminogen

## Abstract

**Introduction:**

Relapsing fever (RF) remains a neglected human disease that is caused by a number of diverse pathogenic *Borrelia* (*B.*) species. Characterized by high cell densities in human blood, relapsing fever spirochetes have developed plentiful strategies to avoid recognition by the host defense mechanisms. In this scenario, spirochetal lipoproteins exhibiting multifunctional binding properties in the interaction with host-derived molecules are known to play a key role in adhesion, fibrinolysis and complement activation.

**Methods:**

Binding of CihC/FbpC orthologs to different human proteins and conversion of protein-bound plasminogen to proteolytic active plasmin were examined by ELISA. To analyze the inhibitory capacity of CihC/FbpC orthologs on complement activation, a microtiter-based approach was performed. Finally, AlphaFold predictions were utilized to identified the complement-interacting residues.

**Results and discussion:**

Here, we elucidate the binding properties of CihC/FbpC-orthologs from distinct RF spirochetes including *B. parkeri*, *B. hermsii*, *B. turicatae*, and *B. recurrentis* to human fibronectin, plasminogen, and complement component C1r. All CihC/FbpC-orthologs displayed similar binding properties to fibronectin, plasminogen, and C1r, respectively. Functional studies revealed a dose dependent binding of plasminogen to all borrelial proteins and conversion to active plasmin. The proteolytic activity of plasmin was almost completely abrogated by tranexamic acid, indicating that lysine residues are involved in the interaction with this serine protease. In addition, a strong inactivation capacity toward the classical pathway could be demonstrated for the wild-type CihC/FbpC-orthologs as well as for the C-terminal CihC fragment of *B. recurrentis*. Pre-incubation of human serum with borrelial molecules except CihC/FbpC variants lacking the C-terminal region protected serum-susceptible *Borrelia* cells from complement-mediated lysis. Utilizing AlphaFold2 predictions and existing crystal structures, we mapped the putative key residues involved in C1r binding on the CihC/FbpC orthologs attempting to explain the relatively small differences in C1r binding affinity despite the substitutions of key residues. Collectively, our data advance the understanding of the multiple binding properties of structural and functional highly similar molecules of relapsing fever spirochetes proposed to be involved in pathogenesis and virulence.

## Introduction

*Borrelia* (*B.*) species transmitted by hematophagous ectoparasites of the genera *Ornithodoros* and *Ixodes* or by the human body louse *Pediculus humanus corporis* are the causative agents of relapsing fever (RF) (1–4). This infectious disease of bacterial origin is characterized by an abrupt onset of high fever followed by two or more recurrent episodes of fever separated by afebrile periods. Depending on the occurrence of the vectors, RF caused by argasid ticks is mainly found in mountainous areas at the West Coast of North America, in south-western and south-central countries of the United States, northern Mexico, and in temperate and tropical regions of different African territories (5, 6). In contrast, louse-borne RF caused by *B. recurrentis* is geographically restricted to countries along the Horn of Africa including Ethiopia, Eritrea, Somalia, and South-Sudan (3). In the US, *Ornithodoros (O.) hermsi* and *O. turicata* are the two main vectors transmitting *B. hermsii* and *B. turicatae*, respectively, both of which have been confirmed to be human pathogens, whereas epidemiological evidence for *B. parkeri* vectored by *O. parkeri* to cause RF in humans has rarely been implicated to date (7).

Once transmitted to the human host during tick feeding, RF spirochetes achieve high cell concentrations in human blood indicating that *Borrelia* species developed means to successfully outwit innate immunity (1). So far, immune evasion strategies developed by RF spirochetes to counteract complement as the first line of defense involves (i) recruitment of endogenous complement regulators (C1-esterase inhibitor (C1-Inh), C4b-binding protein (C4BP), factor H (FH), FH-like protein-1 (FHL-1), factor H-related protein (FHR-1, FHR-2), vitronectin), (ii) interaction with individual complement components (C1r, C3, C3b, C4b, C5) and (iii) degradation of complement components C3 and C5 by activation of surface-bound plasmin(ogen) (1, 8, 9).

Studies aimed at identifying the complement-inhibiting determinants in *B. parkeri*, *B. turicatae*, and *B. hermsii* revealed that these outer surface proteins exhibit considerable diversity in structure and function (10). Among the complement-affecting proteins already described among RF spirochetes, CihC (Complement Inhibition via C4BP) of *B. recurrentis* and *B. duttonii* displays multiple functionalities in terms of inhibiting complement at an early point of activation by binding to C1-Inh inhibitor and C4BP (11). CihC orthologs were also identified in *B. turicatae*, and *B. parkeri* all of which bound to fibronectin but not to complement regulator C1-Inh and C4BP (10). In contrast, a CihC-orthologous protein of *B. hermsii* strain HS1, BHA007 (later on termed FbpC) exhibits a strong affinity to human fibronectin and C1r but binds C4BP to a lesser extent (12, 13). Furthermore, CihC and FbpC share significant sequence homology with BBK32, the first identified fibronectin-binding protein of the Lyme disease causing spirochete *B. burgdorferi* (14, 15). Recently, BBK32 has also been identified as a C1r-binding protein that specifically targets the classical pathway (CP) and promotes resistance of *B. burgdorferi* to complement-mediated killing (16, 17). Phylogenetically, these proteins, including CihC of *B. recurrentis* and *B. duttonii*, are grouped into three distinct subfamilies designated FbpA, FbpB, and FbpC, respectively, whereas CihC clustered with the FbpC-like proteins of other RF spirochetes (12). Unlike *B. burgdorferi*, RF spirochetes including *B. recurrentis*, *B. duttonii*, *B. hermsii*, *B. turicatae*, *B. parkeri*, and *B. miyamotoi* possess up to three distinct fibronectin-binding homologs (10, 11, 13, 18). Despite their sequence homology, they exhibit considerable differences in their ability to interact with fibronectin. For example, FbpA of *B. miyamotoi* retains fibronectin-binding properties, FbpB of *B. miyamotoi* did not (18). In contrast, both proteins exhibit strong affinity to activated C1r, the initiating serine protease of the CP and thereby promote complement inactivation.

In the present study, CihC/FbpC-orthologs from distinct RF spirochetes including *B. parkeri*, *B. turicatae*, *B. hermsii*, and *B. recurrentis* were analyzed regarding their multifunctional binding properties to three host-derived components all of which play a key role in either cell adhesion (fibronectin), fibrinolysis (plasminogen) or CP inactivation (C1r). The data collected herein demonstrate comparable binding properties of CihC/FbpC-orthologs to fibronectin, plasminogen, and C1r, respectively. All borrelial proteins exhibited a strong inactivation property toward the CP and protected serum-susceptible *Borrelia* cells from complement-mediated killing. Taken together, our findings implicate that CihC/FbpC orthologs inherently possess binding properties to diverse host proteins all of which known to play key roles in cell adhesion, bacterial dissemination, and host defense.

## Materials and methods

### Bacterial strains and culture conditions

*B. burgdorferi* B314, a high-passage derivative of reference strain B31 (19) was cultured until mid-exponential phase (5 x 10^7^ cells per ml) at 33°C in Barbour-Stoenner-Kelly (BSK-H) medium (Bio&SELL, Feucht, Germany) supplemented with 7.4% heat-inactivated rabbit serum (Merck, Darmstadt, Germany). Hexahistidine His-tagged proteins were produced in *Escherichia (E.) coli* BL21 (DE3) (New England Biolabs, Frankfurt, Germany), *E. coli* M15 (Qiagen, Hilden, Germany) or *E. coli* C43 (20) grown in yeast tryptone (YT) broth supplemented with ampicillin (50 µg/ml) at 37°C.

### Human serum, proteins, and antibodies

Non-immune human serum (NHS) was collected from healthy volunteers and initially tested for the presence of anti-*Borrelia* IgM and IgG antibodies as described previously (21). Only seronegative samples were combined to form the serum pool and complement activity (CH50) was tested by applying the WIESLAB^®^ Complement Alternative Pathway kit (SVAR Life Science, Malmö, Sweden) according to the manufacturer instructions. Complement factor C1r was purchased from Complement Technology (Tyler, TX, USA), the goat anti-C1r antibody was obtained by bio-techne (Minneapolis, MN, USA), the neoepitope-specific monoclonal anti-C5b-9 antibody was from Quidel (San Diego, CA, USA). Human glu-plasminogen was purchased from Prolytix (Essex Junction, VT, USA) and urokinase plasminogen activator (uPA) (Merck, Darmstadt, Germany) was used for the activation of plasminogen to plasmin. Fibronectin, the anti-fibronectin antibody, chromogenic substrate S-2251 (D-Val-Leu-Lys *p*-nitroanilide dihydrochloride), Zymosan A, and bovine serum albumin (BSA) were obtained from Merck. The polyclonal anti-plasminogen antibody was from Acris Antibodies (Herford, Germany) and gelatin was from Applichem (Darmstadt, Germany). The mouse anti-His antiserum was obtained from Novagen (Merck, Darmstadt, Germany) and Qiagen (Hilden, Germany), and the horseradish peroxidase (HRP)-conjugated immunoglobulins were purchased from Dako (Hamburg, Germany).

### Generation and purification of His-tagged proteins

The generation of His-tagged CihC orthologs from *B. recurrentis* A17, *B. hermsii* FRO, *B. parkeri*, and *B. turicatae* as well as the N- and C-terminal fragment of CihC of *B. recurrentis* A17 and the N-terminal fragment of the CihC ortholog of *B. hermsii* HS1 were described previously (10, 22). The recombinant proteins used herein consisted the following amino acid residues: CihC of *B. recurrentis* (aa 20-356), FbpC of *B. hermsii* FRO (aa 21-374), FbpC ortholog of *B. parkeri* (aa 25-349), FbpC ortholog of *B. turicatae* (aa 21-363), CihC-N of *B. recurrentis* A17 (aa 20-194), CihC-C of *B. recurrentis* (aa 195-356), and FbpC-N from *B. hermsii* (aa 21-203). CspA and BBA70 of *B. burgdorferi* B31 as well as the C-terminal fragment of BBK32 originated from *B. burgdorferi* B31 were used as controls (21, 23, 24).

To produce recombinant proteins, *E. coli* cultures (500 ml) were grown in ampicillin-supplemented TB medium at an OD_600_ of 0.5 to 0.7 and then induced with 200 µM isopropyl-β-D-thiogalactopyranoside (Carl Roth, Karlsruhe, Germany). Cultures were then incubated for 4 h at RT. Following sedimentation, bacterial cells were lysed in buffer containing 300 mM NaCl, 50 mM NaH_2_PO_4_, 10 mM imidazole, and 1 mg/ml lysozyme (Merck, Darmstadt, Germany) (pH 8.0) with a MICCRA D-9 dispersion device (Art Prozess- & Labortechnik, Müllheim, Germany). The cell suspension was thereafter sonicated six times for 30 s at 4°C with a Sonifier 450 (Branson, Danbury, CT, USA). Cell debris were removed by centrifugation and His-tagged proteins were purified in the presence of cOmplete™ protease inhibitor (Roche Diagnostics GmbH, Mannheim, Germany) by affinity chromatography using NEBExpress^®^ Ni Resin (New England Biolabs, Frankfurt, Germany). Increasing concentrations of imidazole (50 to 250 mM) were used for eluation and small amounts of each fraction were loaded on 10% Tris/Tricine SDS-PAGE followed by silver staining. Eluates containing highly purified protein were combined and dialyzed several times against 50 mM Tris buffer and then concentrated by using Protein Concentrator PES (Thermo Scientific, Rockford, IL, USA). The bicinchoninic acid protein assay (Thermo Scientific, Rockford, IL, USA) was used to determine the protein concentration.

### SDS-PAGE, Western and Far-Western blotting

To ensure that no protein degradation occurs during the purification process, His-tagged proteins (500 ng each) were separated by 10% Tris/Tricine SDS-PAGE and visualized by either silver staining or by Western blotting as described previously (23). Briefly, after separation and transfer of the proteins, nitrocellulose membranes were blocked with 5% nonfat dry milk in TBS containing 0.1% Tween 20 (TBS-T1). Following three wash steps with TBS-T1, membranes were incubated with a mixture of monoclonal anti-His antibodies (1:1000) for 1 h at RT. The membranes were washed three times with TBS containing 0.2% Tween 20 (TBS-T2) and incubated with a polyclonal horseradish peroxidase (HRP)-conjugated anti-mouse antibody (1:1000). After washing with TBS-T2, protein complexes were visualized by adding tetramethylbenzidine (TMB) (Mikrogen Diagnostics, Neuried, Germany) as substrate. For Far-Western blotting, membranes were blocked, washed and incubated with fibronectin (5 µg/ml) for 1 h at RT. After washing with TBS-T1, membranes were incubated with an anti-fibronectin antibody (1 h at RT) following incubation with a polyclonal HRP-conjugated anti-mouse antibody. Protein-protein complexes were detected by using TMB.

### Enzyme-linked immunosorbent assay

ELISA was performed to assess binding of complement components C1r, plasminogen or fibronectin to purified His-tagged proteins. Microtiter plates (MaxiSorp, Nunc) were coated with purified recombinant proteins, BSA and gelatin, respectively (5 µg/ml each) at 4°C overnight as described previously (25, 26). Briefly, following blocking with PBS-T containing 0.2% gelatin, C1r (5 µg/ml), plasminogen (10 µg/ml) or fibronectin (10 µg/ml) were added and the plates were incubated for 1 h at RT. Wells were then incubated for 1 h at RT with specific antisera (each diluted 1:1000) followed by an incubation with HRP-conjugated anti-rabbit or anti-goat antisera (1:1000) for 1 h at RT. Afterwards, *o-*phenylenediamine (Merck, Darmstadt, Germany) was added to the wells and the absorbance was measured at 490 nm (PowerWave HT, Bio-Tek Instruments, Winooski, VT, USA). In addition, CihC/FbpC orthologs were immobilized and incubated with increasing amounts of plasminogen (0 to 1.5 µM) to determine dose-dependency of the binding and to calculate the dissociation constant.

### Plasmin(ogen) activation assay

Conversion of protein-bound plasminogen to proteolytically active plasmin was assayed by cleavage of the chromogenic substrate D-Val-Leu-Lys-*p*-nitroanilide dihydrochloride as described previously (24, 25). In brief, microtiter plates coated with purified His-tagged proteins or BSA (5 µg/ml each) in immobilization buffer PBS were blocked with PBS-T-1% BSA for 2 h at RT, washed and then incubated with 10 µg/ml of glu-plasminogen for 1 h at RT. After washing, wells were incubated with TBS-T (50 mM Tris/HCl, pH 7.5, 300 mM NaCl, 0.003% Triton X-100) containing the chromogenic substrate S-2251 (0.3 mg/ml). Finally, 4 µl of 2.5 µg/ml urokinase plasminogen activator (uPA) were added to each well to activate protein-bound plasminogen. Plates were then placed in a spectrophotometer (PowerWave HT) and incubated at 37°C for 24 h. Kinetic measurements were conducted by reading the reactions every 30 minutes at 405 nm. In addition, control reactions containing 50 mM tranexamic acid or in which plasminogen or uPA have been omitted were also measured in parallel.

### Complement inactivation assays

The inhibitory capacity of CihC/FbpC orthologs on the classical (CP) and alternative pathway (AP) was assessed by employing an ELISA-based approach as described previously (26, 27). Microtiter plates were coated with either human IgM (30 ng/ml) (Merck, Darmstadt, Germany) for the CP and Zymosan A (10 µg/µl) (Merck, Darmstadt, Germany) for the AP at 4°C overnight. Following three wash steps with TBS containing 0.05% Triton X-100 (TBS-T), wells were blocked with PBS containing 0.05% Tween20 and 1% BSA for 1 h at RT. NHS (1% for the CP and 15% for the AP) was pre-incubated with increasing concentrations of His-tagged proteins for 15 min at 37°C before added to the wells to initiate complement activation. After washing with TBS-T, the anti-C5b-9 antibody (1:500) (Quidel, Athens, OH; USA) was added to determine assembly of the pore-forming membrane attack complex. Following incubation for 1 h at RT, wells were washed thoroughly with TBS-T and incubated with HRP-conjugated anti-mouse IgG (1:1000) at RT for 1 h. After adding *o-*phenylenediamine, the absorbance values were measured at 490 nm.

### Serum protection assay

Protection of spirochetes from complement-mediated killing by soluble CihC/FbpC orthologs was assessed by a modified serum protection assay as described previously (23). Initially, human serum was incubated with 5 µM of purified CihC/FbpC orthologs or the C-terminal BBK32 fragment as control for 15 min at 37°C with gentle agitation. The reaction mixtures were then adjusted to 100 µl with BSK-H medium. As additional control reactions, native NHS (not pre-treated), heat-inactivated NHS (each 30%) were also included. In parallel, 1 × 10^7^ spirochetes of the serum-sensitive *B. burgdorferi* strain B314 were sedimented by centrifugation and resuspended in the protein-treated serum samples as well as the controls. Spirochetes were then incubated for 4 h at 33°C. For the determination of motile cells, the number of spirochetes was adjusted by various dilutions to 5 up to 25 cells per grid in a single chamber. For every reaction and time point (0, 2, and 4 h), spirochetes in nine grids were counted, and the average was used for the calculation of the total number of cells. The percentage of motile cells was determined by dark field microscopy at time point 0, 2, and 4 h. Biological replicates were conducted at least three times.

### Statistical analysis

The data collected represent means from at least three independent experiments, and error bars indicate SD. For statistical analyses, one-way ANOVA with Dunnett´s multiple comparison post-hoc test (95% confidence interval) was employed for statistical analysis using GraphPad Prism version 10 (GraphPad Software, San Diego, CA, USA). Results were deemed statistically significant for the following p values: *, *P* ≤ 0.05, ** *P* ≤ 0.01 ***, *P* ≤ 0.001, and ****, *P* ≤ 0.0001.

### Structure prediction analyses

The protein sequence of CihC of *B. recurrentis*, FbpC (BHA007) of *B. hermsii* HS1, FbpA (FbpC) of *B. hermsii* FRO, FbpA (FbpC) of *B. parkeri*, and FbpA (FbpC) of *B. turicatae* were used for structural prediction (NCBI accession numbers FN552439, KJ599624.1, HE983605, HE983608) (10–12). After removal of the unfolded N-terminal region, based on Clustal Omega (28) amino acid sequence alignments and the existing structures of *B. miyamotoi* FbpB (7RPS) and FbpA (7RPR), the remaining C-terminal protein sequence was submitted on the AlphaFold2 advanced interface (29) with the default settings using the optional “Refine structures with Amber-Relax” option. Results were exported to UCSF ChimeraX 1.2.5 (30), and modeled using the Matchmaker function for the overlays.

### Ethics statement

Collection of blood samples and consent documents were approved by the ethics committee at the University Hospital of Frankfurt (control number 160/10 and 222/14), Goethe University of Frankfurt am Main. All healthy blood donors provided written informed consent in accordance with the Declaration of Helsinki.

## Results

### CihC/FbpC orthologs of relapsing fever spirochetes bind to fibronectin

Previous investigations revealed interaction of FbpA (hereafter re-designated as FbpC) of *B. hermsii* strains YOR and FRO to fibronectin (10). *In situ* analyses using bioinformatics suggested that FbpC orthologs of *B. parkeri* and *B. turicatae* might also interact with fibronectin, but the authors did not characterize this protein-protein interaction experimentally. In addition, no data are available so far demonstrating binding of fibronectin to CihC of *B. recurrentis* as well. To extend on these previous observations and close the knowledge gap, we conducted ELISA to gather more detailed information on the fibronectin-binding capability of diverse FbpC orthologs including the N- and C-terminal CihC fragments of *B. recurrentis* (CihC-N_Br and CihC-C_Br) and an N-terminal FbpC fragment of *B. hermsii* HS1 (CihC/FbpC-N_Bh-HS1). Hence, FbpC orthologs share high homology to CihC (54%) (10, 11), these molecules were hereafter collectively termed as CihC/FbpC orthologs to avoid misunderstanding due to different nomenclatures used in the literature (see **Supplementary Table 1**).

**Figure 1:**
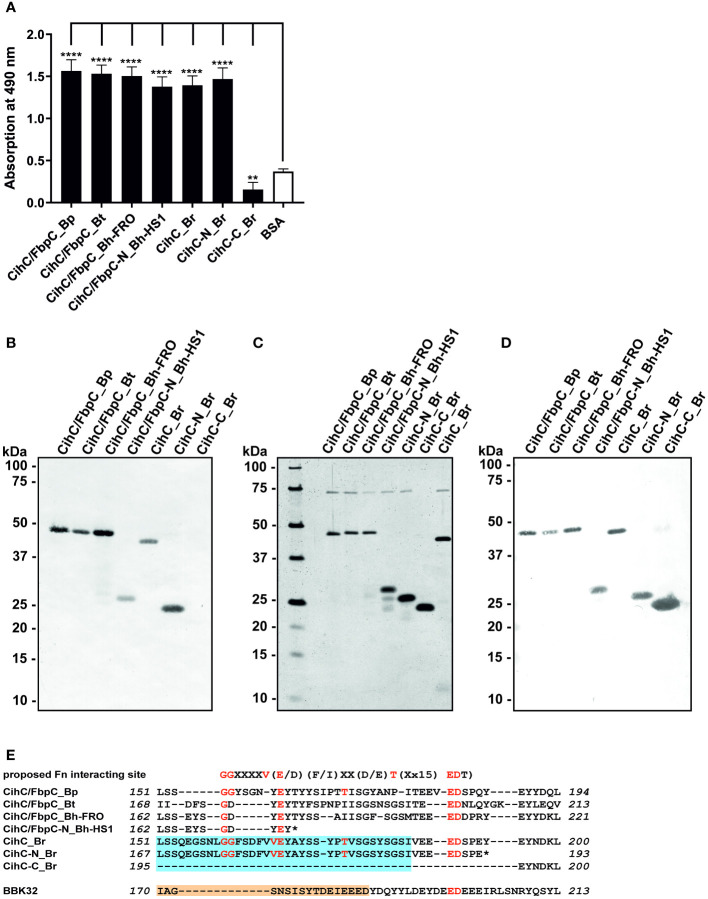
Binding of CihC/FbpC orthologs to fibronectin. **(A)** Binding of fibronectin to recombinant proteins was determined by ELISA. Borrelial proteins or BSA (negative control) (5 µg/ml each) were immobilized and incubated with 10 µg/ml fibronectin. Protein complexes were detected using a monoclonal antibody (1:1000). Data represent means and standard deviation of at least three different experiments, each conducted in triplicate. ****, p ≤ 0.0001, ** p ≤ 0.01, ns, no statistical significance, one-way ANOVA with Dunnett multiple comparison test. **(B)** Binding of fibronectin to purified proteins were detected by Far-Western blot analysis using a monoclonal anti-fibronectin antibody (1:1000). **(C)** Detection of purified proteins by SDS-PAGE following silver staining. **(D)** Western blot analysis with purified proteins employing anti-His antibodies (1:1000). **(E)** Indication of the proposed fibronectin (Fn) binding site as found in diverse human pathogenic bacteria (31). Identical residues of CihC/FbpC orthologs are in red and the putative binding site of C4BP and C1-Inh is boxed (light blue). The fibronectin-binding site identified in BBK32 (32) is indicated in light orange. *, stop codon..

To investigate interaction with fibronectin, microtiter plates were coated with purified proteins and binding was detected. As shown in **Figure 1A**, all CihC/FbpC orthologs and the N-terminal CihC fragments of *B. hermsii* and *B. recurrentis* clearly bound human fibronectin. In contrast, the C-terminal CihC fragment of *B. recurrentis* lacking the predicted binding motif did not interact with fibronectin. To further confirm the data obtained with ELISA, a Far-Western blot analysis was conducted. Again, binding of fibronectin could clearly be demonstrated for all CihC/FbpC orthologs but not to the C-terminal CihC fragment (**Figure 1B**). In addition, silver staining (**Figure 1C**) and Western blotting using anti-His antibodies (**Figure 1D**) were also conducted to demonstrate equal loading and purity of the analyzed His-tagged CihC/FbpC orthologs and variants. The findings gathered from the ELISA and Far-Western blotting are in agreement with previous data demonstrating that the central part of CihC/FbpC of *B. hermsii* HS1 and FRO forms the proposed fibronectin-binding region. In addition, the sequence comparison conducted also revealed that the middle region of the CihC/FbpC orthologs participate in binding of fibronectin but not the C-terminus (**Figure 1E**) (10).

### CihC/FbpC orthologs of relapsing fever spirochetes bind to plasminogen

**Figure 2:**
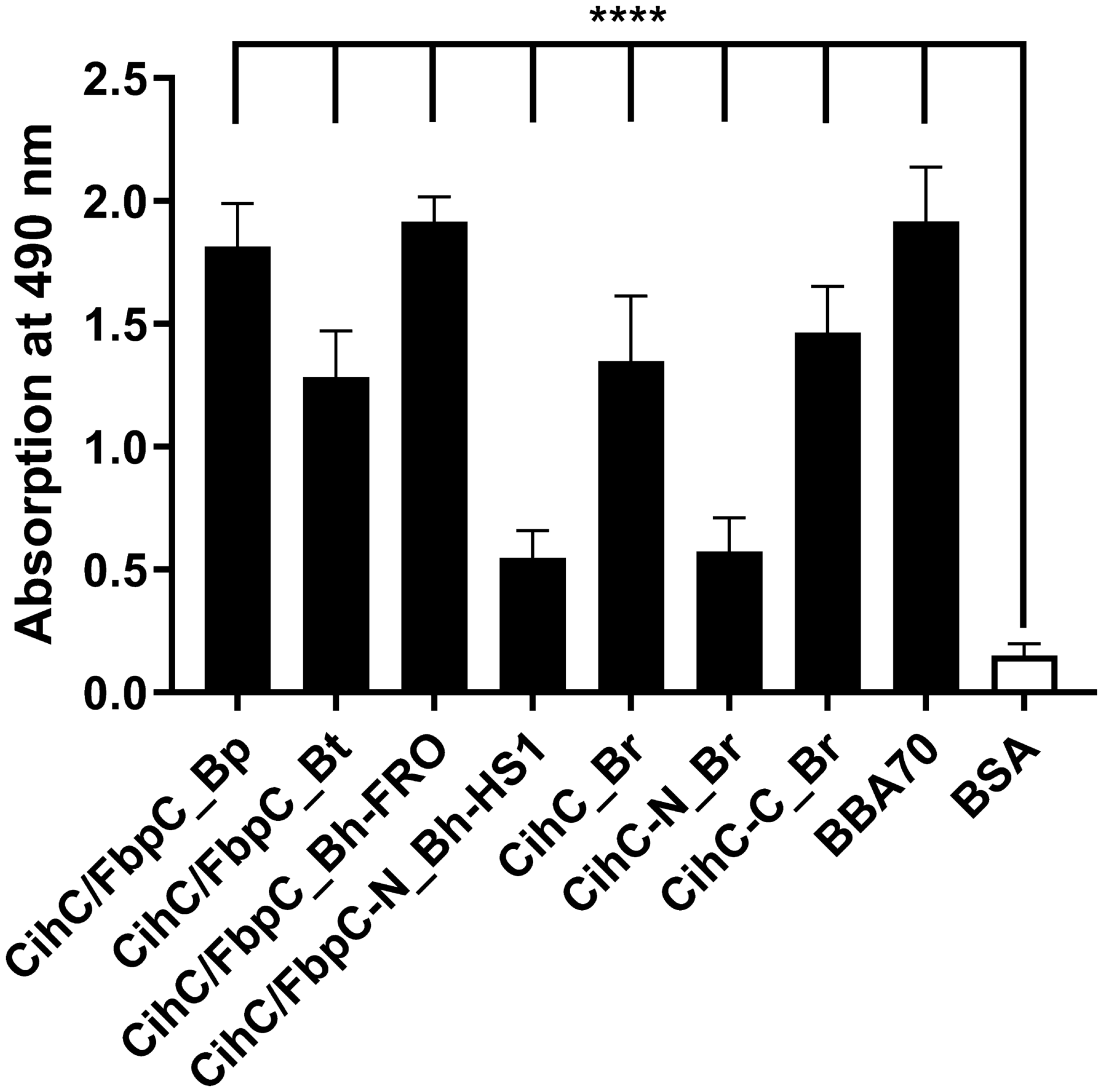
Binding of CihC/FbpC orthologs to plasminogen. Binding of plasminogen to recombinant proteins was determined by ELISA. CihC/FbpC orthologs, N- and C-terminal fragments, BBA70 (positive control) or BSA (negative control) (5 µg/ml each) were immobilized and incubated with 10 µg/ml plasminogen. Protein complexes were detected using a polyclonal anti-plasminogen antibody (1:1000). Data represent means and standard deviation of at least three different experiments, each conducted in triplicate. ****, p ≤ 0.0001, one-way ANOVA with Dunnett multiple comparison test.

It is well known that a number of complement-interacting proteins of Lyme and relapsing fever borreliae act as ligands for the host serine protease plasminogen, allowing spirochetes to disseminate and penetrate to deeper tissues by degrading the key complement factor C3b, as well as extracellular matrix components such as fibrinogen (reviewed in (33–35)). Thus, we examined the ability of CihC/FbpC orthologs to bind human plasminogen by employing ELISA. After immobilization of the proteins, plasminogen was added and protein-protein complexes were detected. The plasminogen-binding protein BBA70 of *B. burgdorferi* (24) and BSA served as positive and negative controls, respectively. Although all proteins including the N-terminal truncated fragments of CihC/FbpC of *B. hermsii* HS1 and CihC of *B. recurrentis* significantly bound to plasminogen when compared to BSA, the truncated proteins appeared to bind to this host protein to a lower extent (**Figure 2**). Overall, our results suggest that plasminogen interacts with CihC/FbpC orthologs at different regions as previously demonstrated for the Factor H-binding proteins BpcA of *B. parkeri*, BtcA of *B. turicatae*, HcpA of *B. recurrentis*, BhCRASP-1 of *B. hermsii*, and CbiA of *B. miyamotoi* (25, 36–38).

**Figure 3:**
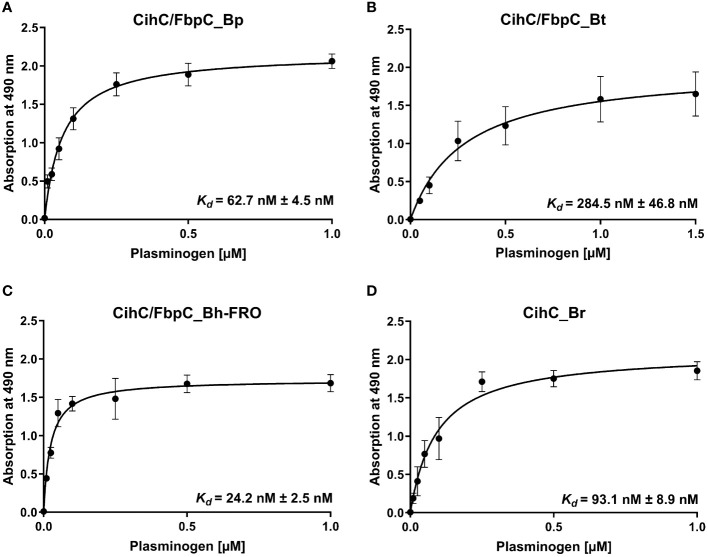
Dose-dependent binding of plasminogen to CihC/FbpC orthologs. Recombinant proteins (5 µg/ml) were immobilized and incubated with increasing concentrations of plasminogen. **(A)**, CihC/FbpC_Bp **(B)**, CihC/FbpC_Bt **(C)**, CihC/FbpC_Bh_FRO **(D)**, CihC_Br. Binding curve and dissociation constant were approximated via non-linear regression, using a one-site, specific binding model. Data represent means and standard deviation of at least three different experiments, each conducted in triplicate.

Having demonstrated binding to plasminogen, we next sought to examine the interaction of CihC/FbpC orthologs of *B. parkeri*, *B. turicatae*, *B. hermsii*, and CihC of *B. recurrentis* with plasminogen in more detail. As depicted in **Figure 3**, a dose-dependent binding of plasminogen to all proteins investigated was apparent whereas CihC/FbpC of *B. hermsii* FRO showed the strongest binding capacity with a calculated apparent dissociation constant of *K_d_* = 24.2 nM ( ± 2.5 nM) following CihC/FbpC of *B. parkeri* [*K_d_* = 62.7 nM ( ± 4.5 nM)], CihC of *B. recurrentis* [*K_d_* = 93.1 nM ( ± 8.9 nM)], and CihC/FbpC of *B. turicatae* [*K_d_* = 284.5 nM (± 46.8 nM)].

### Assessment of the cleavage activity of plasminogen bound to CihC/FbpC orthologs

**Figure 4:**
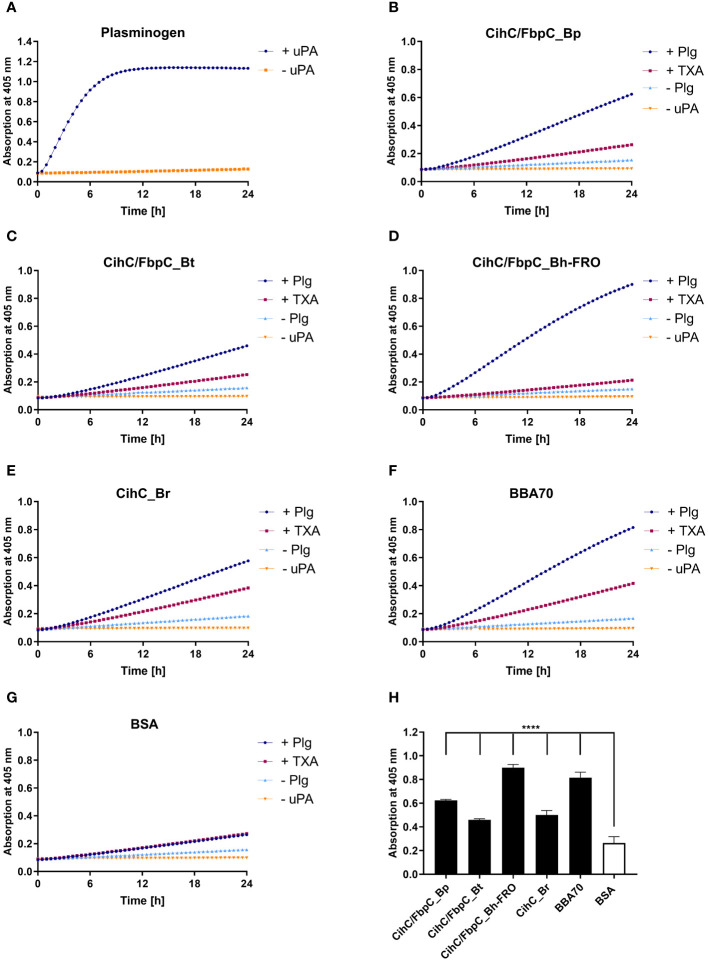
CihC/FbpC-bound plasminogen is converted to active plasmin by uPA. Microtiter plates were coated with 5 µg/ml of plasminogen (Plg) **(A)**, CihC/FbpC_Bp **(B)**, CihC/FbpC_Bt **(C)**, CihC/FbpC_Bh_FRO **(D)**, CihC_Br **(E)**, BBA70 **(F)** or BSA **(G)**. The latter six proteins were subsequently incubated with 10 µg/ml plasminogen. Following several wash steps, a reaction mixture containing the plasminogen activator uPA (final concentration of 0.1 µg/ml) and the chromogenic substrate D-Val-Leu-Lys-p-nitroanilide dihydrochloride (S-2251) was added (●). Control reactions included 50 mM of the lysine analog tranexamic acid (■) or omitted plasminogen (▲) or uPA (▼), respectively. Microtiter plates were incubated at 37°C for 24 h and absorbance at 405 nm was measured at 30 min intervals. At least three independent experiments were conducted, each in triplicate. Data shown are from a representative experiment. Evaluation of the statistical significance **(H)**. The OD values of the final measuring point (24 h) were used for the calculation using GraphPad prism 10. ****) p ≤ 0.0001, one-way ANOVA with Dunnett multiple comparison test.

In circulation, plasminogen is activated by either urokinase-type (uPA) or tissue-type plasminogen activator (tPA) (39). Upon binding, an accessible cleavage site is a prerequisite for plasmin to maintain its proteolytic activity. To demonstrate whether plasminogen bound to CihC/FbpC orthologs is converted to plasmin, proteins were immobilized to microtiter plates and incubated with plasminogen. After adding uPA, cleavage of the plasmin-specific chromogenic substrate D-Val-Leu-Lys-p-nitroanilide dihydrochloride (S-2251) was continuously monitored for up to 24 h. As expected, the strongest proteolytic activity against S-2251 was achieved with purified plasminogen after conversion to plasmin in the presence of uPA (**Figure 4A**). No cleavage occurred when uPA was omitted from the reaction mixtures. By employing CihC/FbpC orthologs, degradation of the chromogenic substrate could be detected by a constant increase of the absorbance values, thus indicating that protein-bound plasminogen was converted to active plasmin (**Figures 4B–E**). A significant increase was also evident for the interaction of plasminogen with BBA70 of *B. burgdorferi*, known to exhibit a strong affinity to plasminogen (55.1 nM) (24) (**Figure 4F**). In contrast, no degradation of the chromogenic substrate occurred when BSA was conducted (**Figure 4G**) or when plasminogen or uPA was omitted to the reaction mixtures (**Figures 4A–G**). Cleavage of the substrate after 24 h was considered statistically significant for CihC/FbpC orthologs of *B. parkeri* and *B. hermsii* FRO, CihC of *B. recurrentis* and the control protein BBA70 (*P* ≤ 0.0001), as well as for CihC/FbpC orthologs of *B. turicatae* (*P* = ≤ 0.0001) (**Figure 4H**).

Considering the participation of lysine residues in the interaction of CihC/FbpC orthologs with plasminogen, tranexamic acid as a lysine analog (40) was added to each reaction mixture. While tranexamic acid strongly inhibited cleavage of the chromogenic substrate when CihC/FbpC orthologs of *B. parkeri*, *B. turicatae*, and *B. hermsii* was assayed, a reduced proteolytic activity of plasmin was observed when CihC of *B. recurrentis* was examined (**Figures 4B–G**). These findings disclose that plasminogen is immediately accessible to uPA and converted to active plasmin upon binding to CihC/FbpC orthologs. Moreover, our data emphasize a prominent role of lysine residues for binding of plasminogen to distinct CihC/FbpC orthologs but also revealed notable differences regarding their interaction with this nonspecific serine protease.

### Determination of the complement-inhibitory activity of CihC/FbpC orthologs

**Figure 5:**
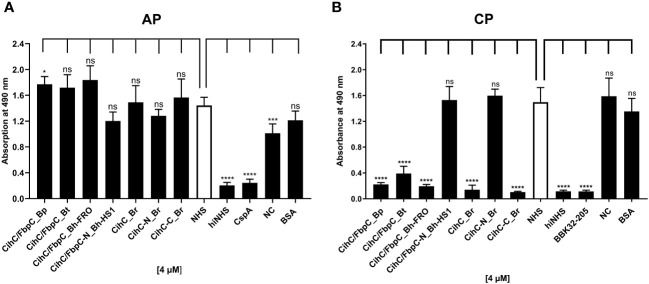
Assessment of the inhibitory properties of CihC/FbpC orthologs on AP and CP activation. ELISA-based functional assays were performed to assess the inhibitory capacity of CihC/FbpC orthologs on the AP **(A)** and CP **(B)**. NHS pre-incubated with the purified CihC/FbpC orthologs, N- and C-terminal fragments, wash buffer (NC) or BSA (4 µM) were added to microtiter plates immobilized with Zymosan A (AP) or IgM (CP). Formation of the MAC was detected by using a monoclonal anti-C5b-9 antibody. Data represent means and standard deviation of at least three different experiments, each conducted in triplicate. ****, p ≤ 0.0001, ***, p ≤ 0.001, *, p ≤ 0.05, ns, no statistical significance, one-way ANOVA with post-hoc Dunnett multiple comparison test (confidence interval = 95%).

**Figure 6:**
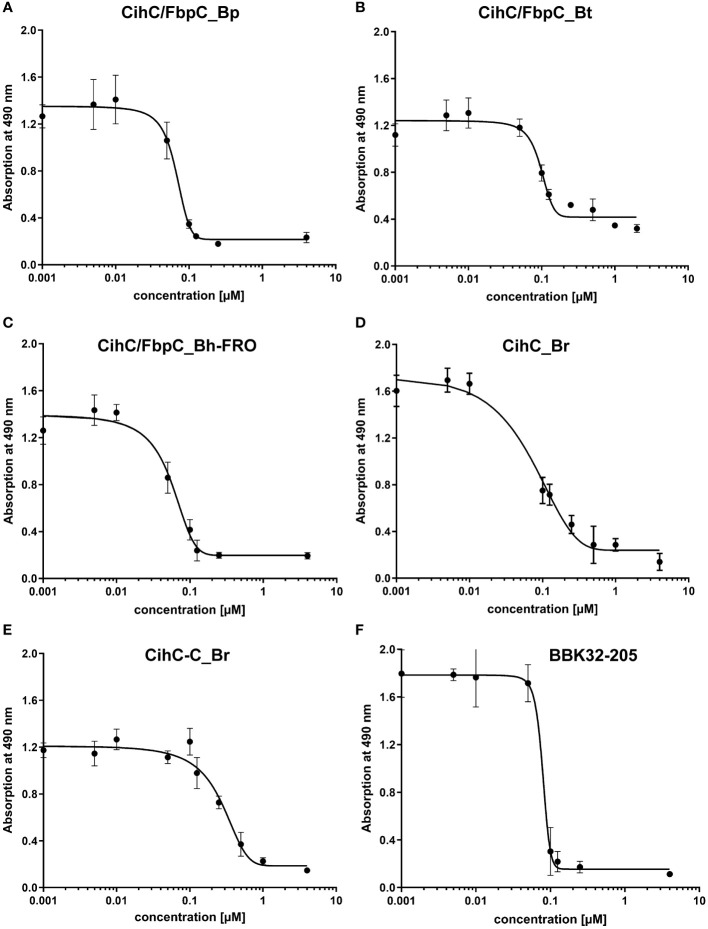
Characterization of the inhibitory capacity of CihC/FbpC orthologs on the CP. Functional assays were performed to characterize the inhibitory capacity of CihC/FbpC orthologs on the CP. NHS pre-incubated with the increasing concentrations (0.001 to 4 µM) of purified CihC/FbpC_Bp **(A)** CihC/FbpC_Bt **(B)** CihC/FbpC_Bh-FRO **(C)** CihC_Br **(D)** CihC-C_Br **(E)**, and BBK32-205 **(F)** were added to microtiter plates immobilized with IgM. Formation of the MAC was detected by using a monoclonal anti-C5b-9 antibody. IC_50_ values were determined using non-linear regression and the one-site, specific binding model. Data represent means and standard deviation of at least three different experiments, each conducted in triplicate.

Previous studies revealed CihC of *B. recurrentis* and FbpC orthologs of *B. miyamotoi* and *B. hermsii* as complement-inhibiting proteins that primarily target the CP at the initial activation step by interacting with complement regulator C1-Inh and complement component C1r, respectively (11, 13). To obtain a closer view in the complement inactivation capacity of these borrelial molecules analyzed, comparative ELISA were performed. As expected, none of the CihC/FbpC proteins affected the AP (**Figure 5A**). In contrast, all CihC/FbpC orthologs and the C-terminal CihC fragment significantly terminated activation of the CP (**Figure 5B**) whereby the N-terminal fragments of *B. recurrentis* and *B. hermsii* as well as BSA (used as control) did not impair complement activation at all. Also, a strong inhibition could be observed when the C-terminal BBK32 fragment of *B. burgdorferi* (16) was employed. Our findings indicate that all CihC/FbpC orthologs displayed similar inactivation capacity toward this particular complement pathway. Next, we sought to elucidate potential differences in the efficacy of these borrelial proteins to inactivate the CP. Our analyses demonstrated that all CihC/FbpC orthologs inhibited this pathway with calculated IC_50_ values ranging between 56 ± 5 nM and 95 ± 11 nM (**Figures 6A–F** and **Table 1**) whereas the C-terminal CihC fragment was less potent in inactivating the CP (272 ± 36 nM).

**Table 1:** CP inhibition (IC_50_).

Protein	*Borrelia* species	IC_50_ (µM)*^b^*
BBK32-205*^a^*	*B. burgdorferi* B31	0.075 ± 0.009
CihC/FbpC	*B. parkeri*	0.063 ± 0.007
CihC/FbpC	*B. turicatae*	0.095 ± 0.011
CihC/FbpC	*B. hermsii* FRO	0.056 ± 0.005
CihC	*B. recurrentis* A17	0.062 ± 0.015
CihC-C*^a^*	*B. recurrentis* A17	0.272 ± 0.036
FbpC-C*^c^*	*B. hermsii* HS1	0.009 ± 0.0008
FbpA-C*^c^*	*B. miyamotoi* FR64b	0.023 ± 0.007
FbpB-C*^c^*	*B. miyamotoi* FR64b	1.900 ± 0.009

^a^C-terminal fragments.

^b^IC_50_ values calculated using a 95% confidence interval.

^c^data collected from (13, 16, 18).

**Figure 7:**
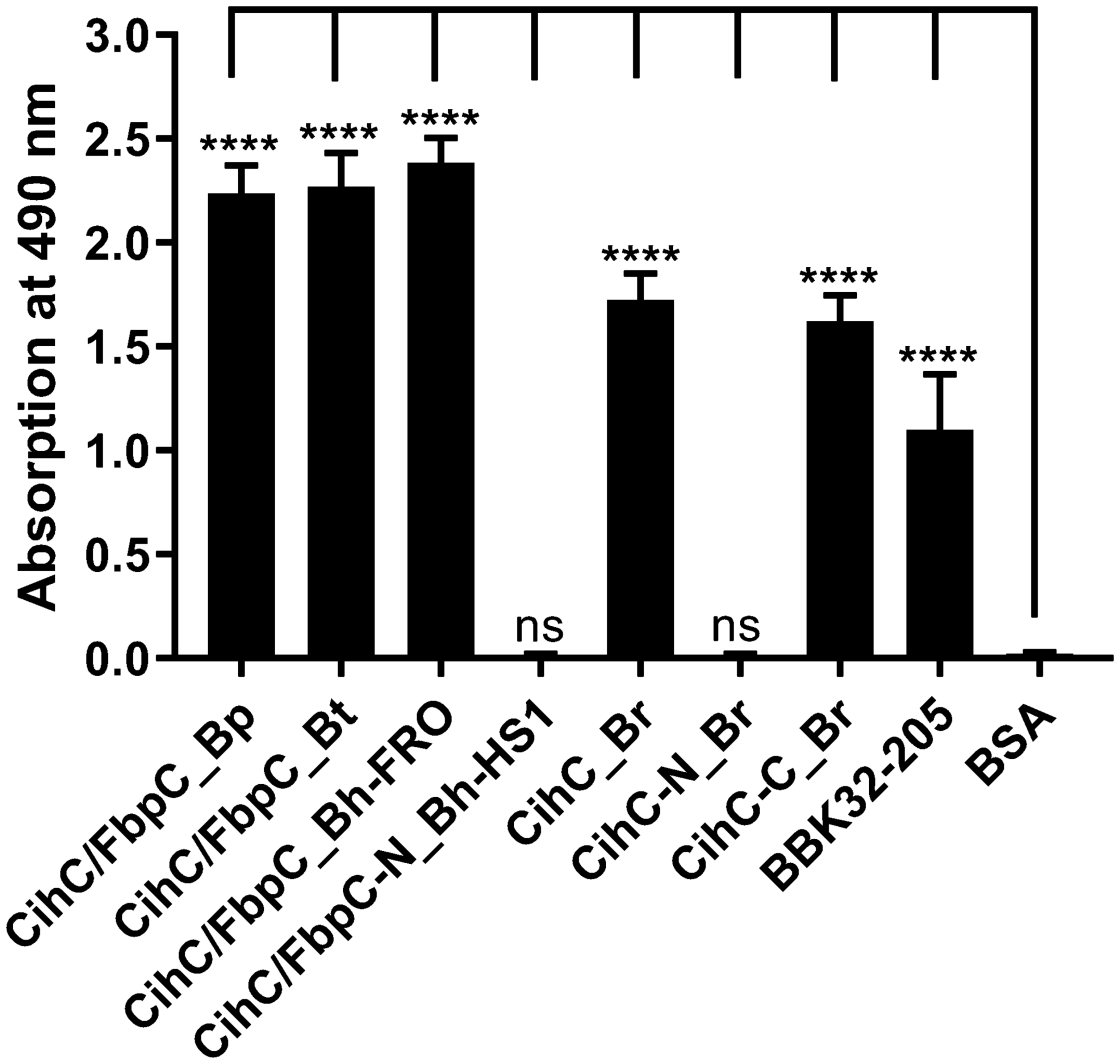
Binding of CihC/FbpC orthologs to C1r. Binding of C1r to recombinant proteins was determined by ELISA. CihC/FbpC orthologs, N- and C-terminal fragments or BSA (negative control) (5 µg/ml each) were immobilized and incubated with 5 µg/ml C1r. Protein complexes were detected using a polyclonal anti-plasminogen antibody (1:1000). Data represent means and standard deviation of at least three different experiments, each conducted in triplicate. ****, p ≤ 0.0001, ns, no statistical significance, one-way ANOVA with Dunnett multiple comparison test.

**Figure 8:**
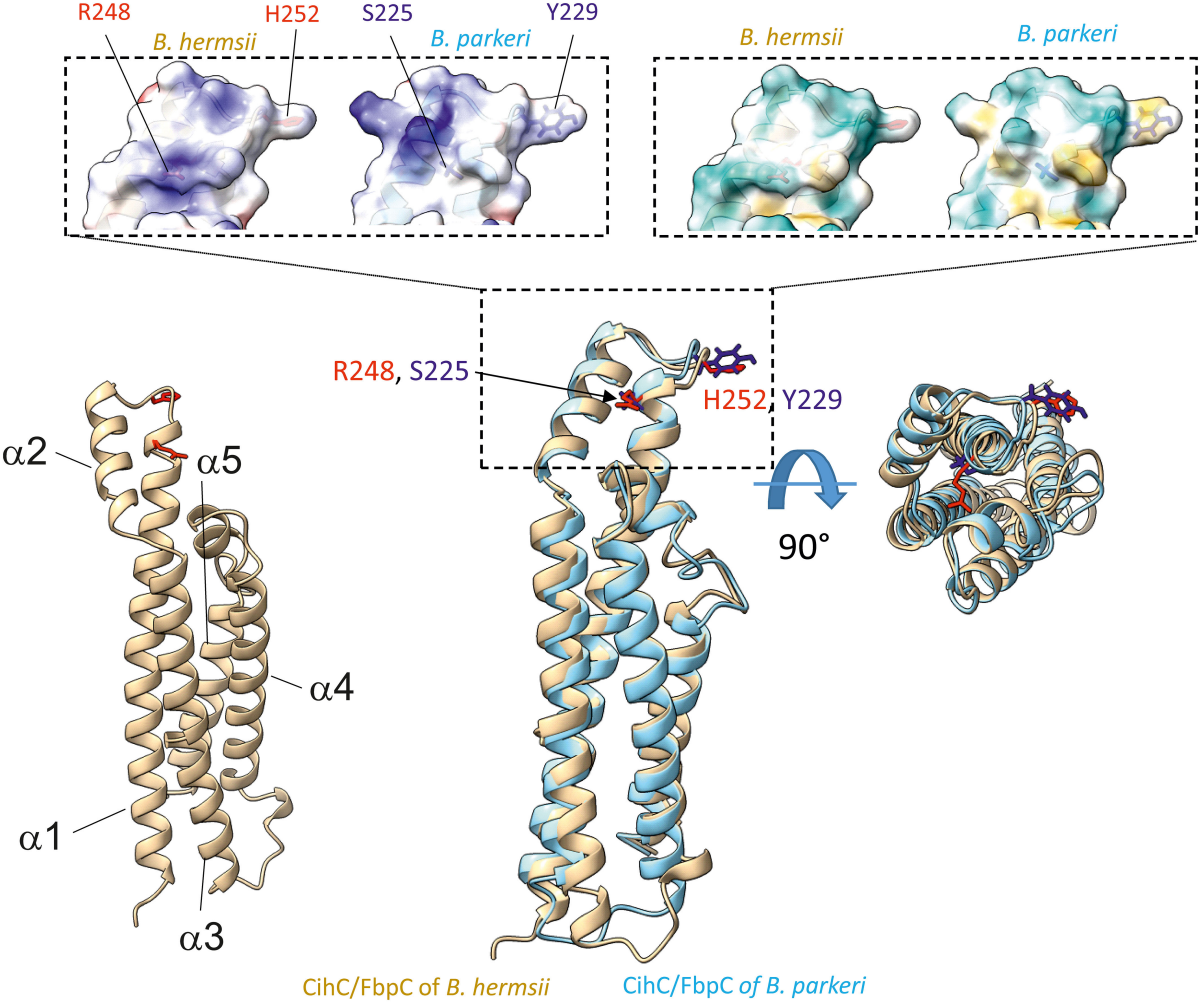
Superimposition of AlphaFold2-predicted structures of *B*. *hermsii* (tan) vs *B*. *parkeri* (sky blue) obtained using the Matchmaker function of ChimeraX. The top of the figure shows the space filling models corresponding to the electrostatic distribution (left inset; positive charges in blue, negative in red) or hydrophobic/hydrophilic residues (right inset; hydrophobic residues in orange/brown, hydrophilic residues in cyan). The residues R248 and H252 from *B*. *hermsii*, located in the α1 helix and the short loop connecting the α1 and α2 helices, respectively, are shown in red, while the matching residues in *B*. *parkeri* are shown in blue. In the bottom right part of the figure, the superimposed ribbon structure was rotated 90° towards the viewer.

As FbpC of *B. miyamotoi* and *B. hermsii* of RF spirochetes and BBK32 of *B. burgdorferi* are known to bind to C1r (13, 16), we wanted to close the gap of knowledge towards the interaction of C1r with CihC of *B. recurrentis* and the CihC/FbpC orthologs of *B. parkeri* and *B. turicatae*. By conducting ELISA, all proteins analyzed significantly bound C1r but the N-terminal CihC/FbpC fragments of *B. hermsii* and *B. recurrentis* did not (**Figure 7**). Our findings are in full agreement with previous data demonstrating that the C-terminal region mediates binding to C1r and also facilitate inactivation of the CP. Moreover, structural comparison of the CihC/FbpC orthologs of *B. hermsii*, *B. parkeri*, *B. turicatae*, and *B. recurrentis* using AlphaFold2 predictions suggest that they adopt a very similar fold, forming an anti-parallel 4-helix bundle (**Figure 8** and **Supplementary Figures 3, 4**). However, despite the strong similarities in the overall folding of the CihC/FbpC proteins from different *Borrelia* strains, their putative C1r binding regions, located in the loop connecting the α1 and α2 helices, has different distributions of charged residues and hydrophobic patches as a result of sequence variation, which may explain differences in binding affinities.

### CihC/FbpC orthologs protect serum-sensitive borrelial cells from complement-mediated lysis

**Figure 9:**
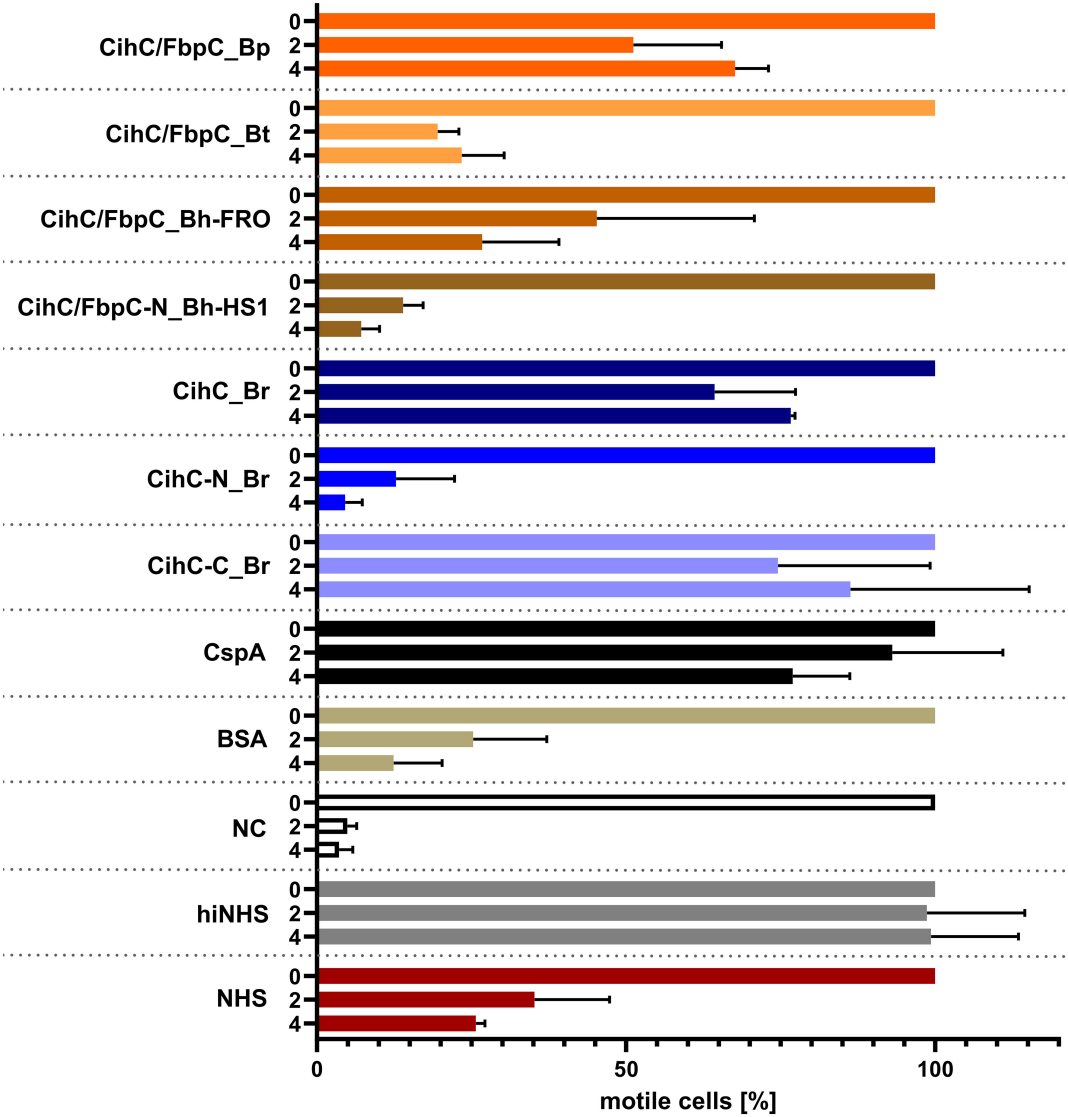
CihC/FbpC orthologs protect serum-sensitive spirochetes from complement-mediated killing. Protection of serum-sensitive *B*. *burgdorferi* B314 by CihC/FbpC orthologs. NHS (30% final) pre-incubated with 5 µM of the respective proteins was added to 1 × 10^7^ spirochetes and viability and motility of borrelial cells were determined at 0, 2, and 4 h of incubation. At least three independent experiments were conducted and ± SEM were calculated.

Previous analyses demonstrated that ectopic expression of CihC of *B. recurrentis*, CihC/FbpC of *B. hermsii* HS1 as well as BBK32 of *B. burgdorferi* in serum-sensitive spirochetes facilitate resistance of these gain-of-function *Borrelia* strains to complement-mediated killing (11, 13). Bactericidal activity of complement could also be efficiently abrogated by pre-incubation of human serum with purified borrelial proteins displaying complement-inactivating properties that leads to the survival of categorically vulnerable spirochetes (13, 16, 18, 23). To further assess the protective nature of the analyzed CihC/FbpC orthologs towards complement, a serum protection assay was conducted by employing all full-length CihC/FbpC orthologs as well as the N- and C-terminal truncated fragments. Initially, native NHS was pre-incubated with purified proteins (5 µM) and then added to viable spirochetes for a final incubation period of 4 h. Almost all CihC/FbpC orthologs including the C-terminal CihC fragment of *B. recurrentis* and the control protein CspA of *B. burgdorferi* conferred protection of susceptible B314 cells from bacteriolysis by human complement (**Figure 9**). However, CihC/FbpC ortholog of *B. turicatae* lacked sufficient inhibitory capacity under these experimental conditions. As expected, heat-inactivated NHS did not influence viability of spirochetes over the whole incubation period. By contrast, spirochetes were efficiently killed in the presence of native NHS or when serum was pre-incubated with the N-terminal CihC/FbpC fragment of *B. hermsii* HS1 and *B. recurrentis*, BSA or with Tris/HCl as buffer control indicating that complement activation was affected by CihC/FbpC orthologs originated from different *Borrelia* species causing relapsing fever.

## Discussion

In this study, we investigated the interaction of key proteins involved in cell adhesion, fibrinolysis, and host defense with CihC/FbpC orthologs from diverse RF spirochetes distributed in North America or Eastern Africa including *B. hermsii*, *B. turicatae*, *B. parkeri*, and *B. recurrentis*, respectively. Although three of these molecules were initially characterized as fibronectin-binding proteins (10), here we sought to elucidate the proposed multifunctional binding properties to two additional host-derived proteins, namely plasminogen and complement factor C1r. Our investigations confirmed that all CihC/FbpC orthologs analyzed bound fibronectin and displayed strong binding affinity to plasminogen. The involvement of lysine residues in the protein-protein interaction was confirmed for three CihC/FbpC orthologs while binding of plasminogen to CihC of *B. recurrentis* appears to be partially lysine dependent. Despite the differences observed, all four molecules promote the conversion of plasminogen to active plasmin in the presence of uPA, and thereby support proteolytic cleavage of the chromogenic substrate. Moreover, our findings disclosed that CihC/FbpC orthologs interacted with C1r and exhibit complement inhibitory properties solely on the CP. More importantly, complement inactivation mediated by these proteins facilitate survival of serum-sensitive spirochetes in the presence of human serum. In summary, the multifunctional binding properties of these particular proteins may enable RF spirochetes to disseminate and overcome initial clearance by the host defence systems as well as to adhere to host cells to promote bacterial extravasation at later time points of the infection.

A common strategy of many human pathogenic microorganisms involves engagement of host-derived, fluid-phase serum proteins to camouflage themselves with, e.g. glycoproteins, inactive precursors, and zymogens to facilitate bacterial adhesion and immune evasion (41–45). One particular strategy of spirochetes involves direct binding to extracellular matrix components, in particular fibronectin and the participation of structural, highly diverse adhesins including BBK32, OspC, RevA, BB0347, CspA, and CspZ of Lyme disease spirochetes (15, 33, 46–53) and the BBK32 orthologs BHA007, FbpA, and FbpC of RF spirochetes (10, 12, 18). Here we show that CihC of *B. recurrentis* and CihC/FbpC orthologs of *B. turicatae* and *B. parkeri* also serve as fibronectin-binding proteins (**Figure 1**). Moreover, the collected data herein provide strong evidence that the fibronectin-interacting region is located in the middle part of these molecules as previously suggested for the FbpC orthologs of *B. hermsii* YOR and HS1, and BBK32 of *B. burgdorferi* B31 (**Figure 1E**) (10, 54, 55). For certain adhesins of diverse human pathogenic bacteria, a putative fibronectin-binding domain with the consensus sequence (GGXXXXV(E/D)(F/I)XX(D/E)T(Xx15)EDT) has been proposed (31). A similar domain could be detected in the central part of CihC of *B. recurrentis* and CihC/FbpC orthologs of *B. turicatae* and *B. parkeri* (**Figure 1E**). By comparison, the predicted binding region appears to be largely inconsistent in BBK32 of *B. burgdorferi* containing an additional repeat of negative-charged aspartate and glutamate residues (EIEEED) (54). Of note, this particular region in CihC also formed the putative binding site for complement regulators C1-Inh and C4BP (11). In contrast to what has previously been published (10), our data revealed that the N-terminal CihC/FbpC fragment of *B. hermsii* HS1 bound to fibronectin likewise the full-length CihC/FbpC orthologs of *B. hermsii* and *B. turicatae* suggesting that a short amino acid stretch engage binding to this particular host protein. As demonstrated earlier (10, 18, 54) and experimentally confirmed here, the C-terminal region lacking the predicted binding domain is not involved in the interaction with fibronectin. Thus, hijacking of soluble fibronectin or direct binding to cellular fibronectin via CihC/FbpC orthologs support adhesion of RF spirochetes to extracellular matrix components and extravasation as previously reported for BBK32 using intra vital microscopy (49, 53, 56–59).

Beside colonization, dissemination through the human host and establishment of an infection is often accompanied with the ability of pathogenic bacteria to recruit soluble activatable precursor molecules from the circulation like plasminogen. This strategy endows human pathogens lacking enzymes with a broad-spectrum proteolytic activity, e.g. borreliae and may increase the invasiveness of those bacteria that are categorically well-equipped with diverse proteases (e.g. *Staphylococcus aureus*, *Streptococcus pyogenes, Candida albicans*) (60–62). Concerning *Borrelia*, it is tempting to speculate that recruitment of plasminogen and conversion to proteolytic active plasmin contribute to the progression of the disease (63–65). Spirochetes produce a number of structural, highly diverse outer surface proteins displaying multiple biological functions. Several studies demonstrated dual binding properties to plasminogen and diverse complement components of certain *Borrelia* proteins including BhCRASP-1, HcpA, BpcA, and CbiA of RF spirochetes as well as OspC, CspA, CspZ, ErpA, ErpP, ErpC, and Erp63 of LD spirochetes (21, 25, 36, 38, 48, 66–69). Here, we present empirical evidence that CihC of *B. recurrentis* and the CihC/FbpC orthologs of RF spirochetes act as ligands for plasminogen as well (**Figure 2**). Plasminogen itself does not solely interact with components of the fibrinolytic system, but also with functionally diverse proteins of human pathogenic microorganisms, mainly via lysine binding pockets localized in the five kringle domains (21, 24, 25, 70–74). Considering the interplay with bacterial proteins, lysine-rich regions at the C-terminus were attributed with the plasminogen-binding activity of enolase of *Streptococcus pneumoniae*, CipA of *Acinetobacter baumannii*, and BBA70 of *B. burgdorferi* (72–74). Intriguingly, a cluster of lysine residues can be found at the C-terminus in CihC of *B. recurrentis* and *B. duttonii*, the CihC/FbpC orthologs of *B. parkeri*, *B. turicatae*, and *B. hermsii* as well as ErpA, ErpC, ErpP, and BBA70 of *B. burgdorferi* (**Supplementary Figure 1**). The reduced plasminogen binding capacity of the N-terminal CihC/FbpC fragments underscore the relevance of the C-terminus to form the key interacting region of these borrelial proteins. However, lacking of C-terminal lysine residues does not mean per se a restricted plasminogen binding capability as previously shown for BhCRASP-1, HcpA, BpcA, and CbiA of RF spirochetes and for OspA, OspC, CspA, and CspZ of *B. burgdorferi*. Our findings support the notion that lysine residues play a substantial role in protein-protein interaction as tranexamic acid significantly abolish binding of plasminogen to CihC/FbpC orthologs of RF spirochetes as previously reported for enolase of *S. pneumoniae* (74), elongation factor Tuf and CipA of *A. baumannii* (72, 73), BBA70 of *B. burgdorferi* (24), CbiA of *B. miyamotoi* (25), and BpcA of *B. parkeri* (38). Obviously, the biological relevance for the acquisition of an inactive precursor molecule has been demonstrated in this study by elucidating the activation of protein-bound plasminogen to plasmin following cleavage of a chromogenic substrate. Hence, spirochetes have the ability to camouflage with a broad-spectrum protease, enabling these primarily blood-borne pathogens to degrade extracellular matrix proteins, thus facilitating invasion and dissemination to various organs in the human host.

**Figure 10:**
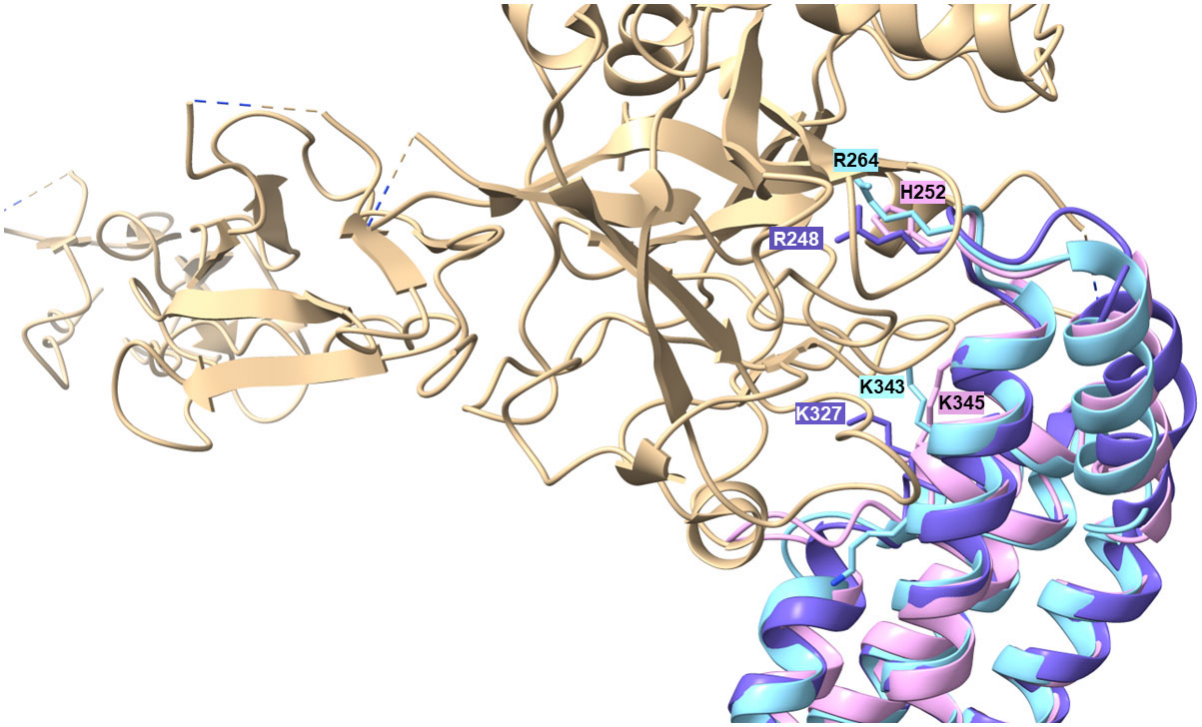
Modelling of α1/α2 loop region into the C1r binding site. The available 7RPR structure of *B*. *miyamotoi* FbpA (sky blue) and the structural AlphaFold2 prediction for *B*. *hermsii* CihC/FbpC (pink/plum) were modelled onto the BBK32 (purple)/C1r (tan) co-crystal structure (7MZT.pdb). The Figure also shows the additional K327 residues described to play a role in BBK32 binding to C1r (75) and the corresponding K343 (*B. myamotoi* FbpA) and K345 (*B. hermsii* CihC/FbpC) positions in the orthologous structures.

Production of proteins displaying multiple binding properties for host-derived proteins involved in key processes such as adhesion, activation of precursor molecules or recognition and elimination of invading microorganisms are beneficial for human pathogenic microorganisms to affect different defense mechanisms of the host simultaneously. For example, dual binding of plasminogen and diverse complement components has previously been described for several outer surface proteins of RF spirochetes including BhCRASP-1 of *B. hermsii*, BpcA of *B. parkeri*, CbiA of *B. miyamotoi*, and HcpA of *B. recurrentis* (10, 25, 37, 38, 67). Later on, multifunctional binding has been elucidated for FbpA of *B. miyamotoi* and FbpC of *B. hermsii* both of which interact with fibronectin and activated C1r (13, 18). These authors demonstrated binding to C1r via an alpha helical domain located at the C-terminus of both proteins as previously reported for BBK32 of *B. burgdorferi* (16). Two residues located in the proposed binding site (R248 and H252 in FbpC of *B. hermsii*, R264 and K343 in FbpA of *B. miyamotoi*, R248 and K327 in BBK32) have been identified to be critical in mediating interaction with C1r (13, 16) (**Supplementary Figure 2**). It has been shown that replacement of either arginine-248 or histidine-252 have a negative impact on the complement inhibitory potential of both FbpC variants. The functional analyses revealed that substitution of histidine-252 by alanine results in a reduced binding capacity to C1r and a decreased inhibitory potential on the CP while replacement of arginine have a lesser effect on C1r binding and complement inhibition. These authors concluded that the so-called dynamic residues in the flexible loop region connecting alpha helices 1 and 2 forms the most important structural part involved in inactivation of the CP. Previous investigations convincingly showed that arginine at position 248 in the prototypic fibronectin-binding protein BBK32 of *B. burgdorferi* is crucial for C1r binding and complement inhibition as well (75). Interestingly, these two key residues are also present in CihC of *B. recurrentis* and *B. duttonii* but could not been found in CihC/FbpC orthologs of *B. parkeri* and *B. turicatae* where arginine is replaced by serine in both proteins and histidine by tyrosine in CihC/FbpC in *B. parkeri*, respectively (**Supplementary Figure 2**). Seemingly, mutations at this particular position do not necessarily have to be accompanied with a loss of C1r binding or complement inhibitory properties of the protein. To understand this observation, we compared the putative C1r-binding loops of the different *Borrelia* CihC/FbpC proteins. As stated above, despite the similarity in the overall fold, this region varies in terms of distribution of charges and hydrophobicity, which may explain differences in binding affinities (**Table 1** and **Supplementary Figures 3, 4**). The variations in the key positions of the C1r binding loop include changes from a positively charged arginine residue to a serine (*B. hermsii* versus *B. parkeri* and *B. turicatae*) or positively charged histidine to an aromatic tyrosine (*B. hermsii* versus *B. parkeri*). Despite these changes, the measured differences in C1r binding appear relatively small (**Figures 6A–F**). This perhaps unexpected finding prompted us to take a closer look at the predicted positions of these key residues R248 and H252 in relation to the C1r binding partner. For this purpose, we modeled the structures of *B. miyamotoi* FbpA (7RPR.pdb) and *B. hermsii* CihC (AlphaFold2 prediction) on the available co-crystal structure of C1r with *B. burgdorferi* BBK32 (7MZT.pdb) (75) (**Figure 10**). The overlay of the existent and predicted three-dimensional structures suggests that the R248, R264 and H252 side chains of *B. burgdorferi* BBK32, *B. miyamotoi* FbpA and *B. hermsii* CihC, respectively, all located in the α1/α2 connecting loop, lie in a similar position within the same binding pocket in C1r, perhaps explaining the similar binding affinities. However, in the absence of a co-crystal structure, the exact contribution of the key residues to C1r binding in the different CihC/FbpC proteins remains to be assessed experimentally.

Our data suggested that the variations in the proposed C1r binding region either did not drastically influence binding of CihC/FbpC orthologs of *B. parkeri* and *B. turicatae* to C1r nor their complement inhibitory properties (**Figures 6**, **7**). The calculated IC_50_ values for the CihC/FbpC orthologs analyzed in this study are in the same range compared to the IC_50_ values of BBK32 of *B. burgdorferi*, FbpA and FbpB of *B. miyamotoi*, and FbpC of *B. hermsii*, respectively (**Table 1**) (13, 16). Deletions and mutations might change the native conformation of a protein following partial or complete loss of the biological function. Concerning CihC/FbpC orthologs, the utilization of C-terminal fragments did not affect their binding to C1r or impact their complement inhibitory properties as recently shown for these CihC/FbpC orthologs. However, the C-terminal fragment of CihC displayed a 4-fold lower inhibitory activity on the CP. Additional structural elements are involved in stabilizing binding of C1r or promoting interaction with complement regulators C1-Inh and C4BP known to be additional ligands of CihC (11). Thus, it is tempting to speculate that proteins exhibiting multiple binding properties, in particular to diverse complement proteins, affect complement activation in a stronger way as shown for CihC. Previous investigations emphasize the importance of these immune evasion molecules for complement resistance of RF spirochetes (11, 13). These *in vitro* studies convincingly demonstrated that serum-sensitive spirochetes ectopically producing CihC of *B. recurrentis* (11), FbpA and FbpB of *B. miyamotoi* (18) or FbpC of *B. hermsii* (13) on their surface are able to resist complement-mediated killing. Likewise, protection from complement-mediated killing could also been achieved when serum was treated with purified proteins before spirochetes were challenged as previously shown for diverse complement-inactivating proteins of LD and relapsing fever spirochetes including the CihC/FbpC orthologs studied herein (13, 16, 18, 23). However, we note that CihC/FbpC ortholog of *B. turicatae* failed to sufficiently protect serum-susceptible B314 from complement-mediated bacteriolysis. This finding could be explained by the weaker complement inhibitory property on the CP and binding affinity to C1r (**Figures 5**, **6** and **Table 1**).

Equipped with complement-binding proteins specifically interacting to certain components of the CP and AP such as Factor H, C4BP, C1-Inh, C1q and C1r, spirochetes more likely overcome the first line of host defense in many ways when the humoral response is a step behind in recognizing and targeting blood-borne pathogens.

In summary, we characterized the multifunctional binding properties of CihC/FbpC orthologs originated from relapsing fever spirochetes to distinct serum-derived proteins involved in adhesion, dissemination and immune evasion. All analyzed proteins displayed remarkable binding properties to fibronectin, plasminogen and complement component C1r. By investigating complement inhibition, these proteins strongly affect activation of the CP and contribute to spirochetes` survival. Elucidating the molecular principles of how relapsing fever spirochetes circumvent host defense mechanisms largely improve our general understanding of the pathological processes in the human host.

## Data availability statement

The raw data supporting the conclusions of this article will be made available by the authors, without undue reservation.

## Ethics statement

The studies involving humans were approved by Ethics committee at the University Hospital of Frankfurt, Goethe University of Frankfurt am Main. Control numbers: 160/10 and 222/14. The studies were conducted in accordance with the local legislation and institutional requirements. The participants provided their written informed consent to participate in this study.

## Author contributions

A-SD: Writing – review & editing, Writing – original draft, Visualization, Validation, Methodology, Investigation, Formal analysis, Data curation, Conceptualization. FR: Writing – review & editing, Writing – original draft, Visualization, Validation, Methodology, Investigation, Formal analysis, Data curation. LL: Writing – review & editing, Writing – original draft, Visualization, Validation, Investigation, Formal analysis, Data curation. Y-PL: Writing – review & editing, Writing – original draft, Resources. FF: Writing – review & editing, Writing – original draft, Visualization, Software, Investigation, Formal analysis, Data curation. PK: Resources, Writing – review & editing, Writing – original draft, Visualization, Validation, Supervision, Project administration, Methodology, Investigation, Funding acquisition, Formal analysis, Data curation, Conceptualization.
