# Supplementary material for: Multifunctional interaction of CihC/FbpC orthologs of relapsing fever spirochetes with host-derived proteins involved in adhesion, fibrinolysis, and complement evasion
Source: Front Immunol. 2024 Apr 25;15:1390468. doi: 10.3389/fimmu.2024.1390468 (PMC11079166; doi:10.3389/fimmu.2024.1390468)
Supplement: Supplementary file 1 [file DataSheet_1.pdf]

Supplementary Table 1. Selected characteristics of CihC/FbpC orthologs of relapsing fever *Borrelia*

Original designation	Categorized by phylogeny	Gene locus[a]	Proposed designation	*Borrelia* species	Isolate	Binding properties[b]	Accession number	Reference	Binding properties[c]
CihC	FbpC	n.d.	CihC	*B. recurrentis*	A1	C1-Inh<br>C4Bp	FN552439	[1]	Fibronectin<br>Plasminogen<br>C1r
FbpA	FbpC	n.d.	CihC/FbpC	*B. parkeri*	RML	Fibronectin	HE983607	[2]	Plasminogen<br>C1r
FbpA	FbpC		CihC/FbpC	*B. turicatae*	91E135 or Oz1	Fibronectin	HE983608	[2]	Plasminogen<br>C1r
FbpC	FbpC	*bha007*	CihC/FbpC	*B. hermsii*	HS1	Fibronectin<br>C4Bp<br>C1r	HE983606	[3]<br>[3]<br>[4]	Plasminogen
FbpA	FbpC		CihC/FbpC	*B. hermsii*	FRO	Fibronectin<br>C4Bp	HE983605	[2]	Plasminogen<br>C1r

[a], loci classified by comparative genomic analyses [5]
[b], binding properties reported from previous investigations
[c], findings reported in this study
n.d., not determined

References:

1. Grosskinsky S, Schott M, Brenner C, Cutler SJ, Simon MM, Wallich R. Human complement regulators C4b-binding protein and C1 esterase inhibitor interact with a novel outer surface protein of *Borrelia recurrentis*. PLoS Negl Trop Dis. 2010;4(6):e698.
2. Brenner C, Bomans K, Habicht J, Simon MM, Wallich R. Mapping the ligand-binding region of *Borrelia hermsii* fibronectin-binding protein. PLoS One. 2013;8(5):e63437. doi: 10.1371/journal.pone.0063437.
3. Lewis ER, Marcsisin RA, Campeau Miller SA, Hue F, Phillips A, Aucoin DP, et al. Fibronectin-binding protein of *Borrelia hermsii* expressed in the blood of mice with relapsing fever. Infect Immun. 2014;82(6):2520-31. doi: 10.1128/IAI.01582-14.
4. Roy S, Booth CE, Jr., Powell-Pierce AD, Schulz AM, Skare JT, Garcia BL. Conformational dynamics of complement protease C1r inhibitor proteins from Lyme disease- and relapsing fever-causing spirochetes. J Biol Chem. 2023;299(8):104972. doi: 10.1016/j.jbc.2023.104972.
5. Kneubehl AR, Lopez JE. Comparative genomics analysis of three conserved plasmid families in the Western Hemisphere soft tick-borne relapsing fever borreliae provides insight into variation in genome structure and antigenic variation systems. Microbiol Spectr. 2023;11(5):e0089523. doi: 10.1128/spectrum.00895-23.
