# Supplementary material for: Multifunctional interaction of CihC/FbpC orthologs of relapsing fever spirochetes with host-derived proteins involved in adhesion, fibrinolysis, and complement evasion
Source: Front Immunol. 2024 Apr 25;15:1390468. doi: 10.3389/fimmu.2024.1390468 (PMC11079166; doi:10.3389/fimmu.2024.1390468)
Supplement: Supplementary file 2 [file Presentation_1.pdf]

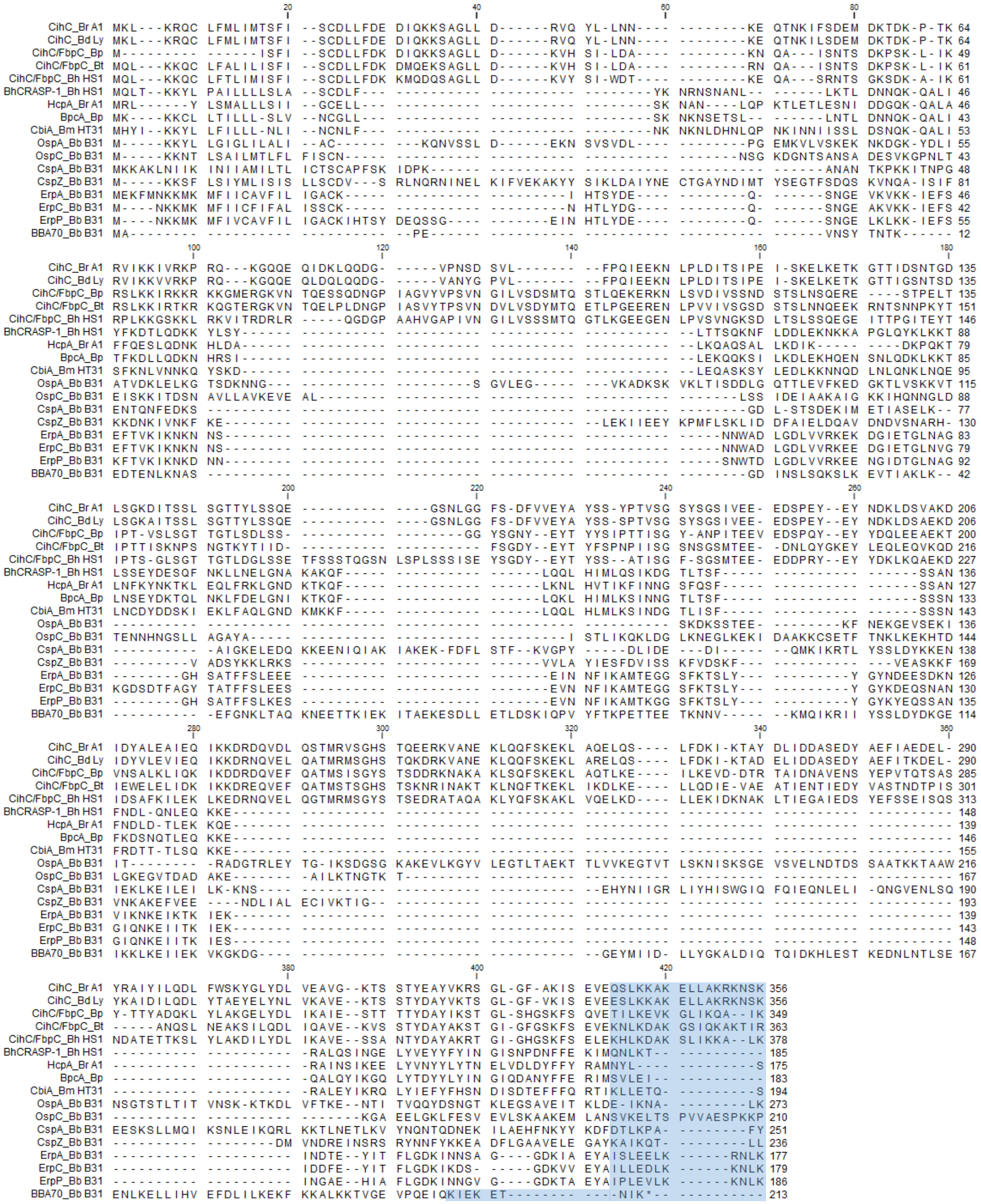

Supplementary figure 1. Sequence alignment of plasminogen-binding proteins of Lyme disease and relapsing fever borreliae. For sequence analyses, the CLC sequence Viewer 8.0 (QIAGEN Aarhus A/S, Denmark) and EMBOSS Needle (https://www.ebi.ac.uk) was used. The proposed C-terminal plasminogen-binding regions are highlighted in light blue.
