# Supplementary material for: Multifunctional interaction of CihC/FbpC orthologs of relapsing fever spirochetes with host-derived proteins involved in adhesion, fibrinolysis, and complement evasion
Source: Front Immunol. 2024 Apr 25;15:1390468. doi: 10.3389/fimmu.2024.1390468 (PMC11079166; doi:10.3389/fimmu.2024.1390468)
Supplement: Supplementary file 3 [file Presentation_2.pdf]

```
                                                20                                      40                                      60                                      80
                                                 |                                       |                                       |                                       |
CihC/FbpC_Bp        M--------- ----ISFISC DLLFDKDIQK KSAGLLDKVH SILDAKNQAI SNTSDKPSKL IKRSLKKIRK KRKKGMERGK 67
CihC/FbpC_Bt        MQ-LKKQCLF ALILISFISC DLLFDKDMQE KSAGLLDKVH SILDARNQAI SNTSDKPSKL IKRSLKKIRT KRKQGTERGK 79
CihC/FbpC_Bh-FRO    MQ-LKKQCLF TLIMISFISC DLLFDKKMQD QSVGLLDKVY SIWDTKEQAS RNTSGKPDKA IKKPLKKGSK KLRKVITRDK 79
CihC/FbpC-N_Bh-HS1  ---------- ---------- DLLFDKKMQD QSVGLLDKVY SIWDTKEQAS RNTSGKPDKA IKKPLKKGSK KLRKVITRDK 60
CihC_Br             MK-LKRQCLF MLIMTSFISC DLLFDEDIQK KSAGLLDRVQ YLLNNKEQTN KIFSDEMDKT DKPTKRVIKK IVRKPRQKGQ 79
CihC-N_Br           ---------- ---------- DLLFDEDIQK KSAGLLDRVQ YLLNNKEQTN KIFSDEMDKT DKPTKRVIKK IVRRPRQKGQ 60
CihC-C_Br           ---------- ---------- ---------- ---------- ---------- ---------- ---------- ---------- -
BBK32               MKKVKSKYLA LGLLFGFISC DLFIRYEMKE ESPGLFDKGN SILETSEESI KKPMNKKGKG KIARKKGKSK VSRKEPYIHS 80

                                               100                                     120                                     140                                     160
                                                 |                                       |                                       |                                       |
CihC/FbpC_Bp        VNTQESSQDN GPIAGVYVPS VNGILVSDSM TQSTLQEKER KNLSVDIVSS NDSTSLNSQE RE----STPE LTIPT-VSLS 142
CihC/FbpC_Bt        VNTQELPLDN GPIASVYTPS VNDVLVSDYM TQETLPGEER ENLPVVIVSG SDSTSLNNQE EKRNTSNNPK YTIPTTISKN 159
CihC/FbpC_Bh-FRO    LR-----QAN GSAAYVGAPI VNGILVSSSM TQGTLKGEEG ENLSVSVNGG NDLTSLSNQE GE---ITAPG ST-PTS-GLS 149
CihC/FbpC-N_Bh-HS1  LR-----QGN GSAAYVGAPI VNGILVSSSM TQGTLKGEEG ENLSVSVNGG NDLTSLSNQE GE---ITAPG ST-PTS-GLS 130
CihC_Br             QEQIDKLQQD G------VPN SDSVL----- ----FPQIEE KNLPLDITSI PEISKELKE- -------TKG TTIDSNTGDL 136
CihC-N_Br           QEQIDKLQQD G------VPN SDSVL----- ----FPQIEE KNLPLDITSI PEISKELKE- -------TKG TTIDSNTGDL 117
CihC-C_Br           ---------- ---------- ---------- ---------- ---------- ---------- ---------- ---------- -
BBK32               LKRDSANKSN -------FLQ KNVIL----- --------EE ESLKTELL-- KEQSETRKEK IQKQQDEYKG MTQGSLNSLS 138

                                               180                                     200                                     220                                     240
                                                 |                                       |                                       |                                       |
CihC/FbpC_Bp        GTTGTLSDLS ---------- ---------S GGY-SGNYEY TYYSIPTTIS GY-ANPITEE VEDSPQY--E YYDQLEEAEK 199
CihC/FbpC_Bt        PSNGTKYTII ---------- ---------D --F-SGDYEY TYFSPNPIIS GSNSGSMTEE --DNLQYGKE YLEQLEQVKQ 215
CihC/FbpC_Bh-FRO    GTTGTLGSLS GKISSYSTKG SNSSPLSGLS SEY-SGDYEY TYSS--AIIS GF-SGSMTEE -EDDPRY--E YYDQLKKAEK 222
CihC/FbpC-N_Bh-HS1  GTTGTLGSLS GKISSYSTKG SNSSPLSGLS SEY-SGDYEY TYS*------ ---------- ---------- ---------- 173
CihC_Br             SGKDITSSLS GTTYLSSQEG SN------LG GFS-DFVVEY AYSSYPT-VS GSYSGSIVEE -EDSPEY--E YNDKLDSVAK 205
CihC-N_Br           SGKDITSSLS GTTYLSSQEG SN------LG GFS-DFVVEY AYSSYPT-VS GSYSGSIVEE -EDSPE*--- ---------- 175
CihC-C_Br           ---------- ---------- ---------- ---------- ---------- ---------- ---------E YNDKLDSVAK 11
BBK32               GESGELEEPI ESNEIDLTID SDLRPKSSLQ GIAGSNSISY TDEIEEEDYD QYYLDEYDEE DEEEIRLSNR YQSYLEGVKY 218

                                               260    R248 H252                        280                                     300                                     320
                                                 |                                       |                                       |                                       |
CihC/FbpC_Bp        TVNSALKLIQ KIKDDRDQVE FQATMSISGY STSDDRKNAK AKLSQFSKEK LAQTLKEILK EVD-DTRTAI DNAVENSYEP 278
CihC/FbpC_Bt        DIEWELELID KIKKDREQVE FQATMSTSGH STSKNRINAK TKLNQFTKEK LIKDLKELLQ DIE-VAEATI ENTIEDYVAS 294
CihC/FbpC_Bh-FRO    DIDSAFKILE KLKKDRDQVE LQGTMRMSGH STSEDRATAQ AKLNQFSKAK LVQELKDLLE KIDKNAKLTI DNAVEDFSKF 302
CihC/FbpC-N_Bh-HS1  ---------- ---------- ---------- ---------- ---------- ---------- ---------- ---------- 173
CihC_Br             DIDYALEAIE QIKKDRDQVD LQSTMRVSGH STQEERKVAN EKLQQFSKEK LAQELQSLFD KI-KTAYDLI DDASEDYAEF 284
CihC-N_Br           ---------- ---------- ---------- ---------- ---------- ---------- ---------- ---------- 175
CihC-C_Br           DIDYALEAIE QIKKDRDQVD LQSTMRVSGH STQEERKVAN EKLQQFSKEK LAQELQSLFD KI-KTAYDLI DDASEDYAEF 90
BBK32               NVDSAIQTIT KIYNTYTLFS TKLTQMYSTR LDNFAKAKAK EEAAKFTKED LEKNFKTLLN YIQVSVK--- ---------- 285
                                                        R248

                                               340                                     360                                     380
                                                 |                                       |                                       |
CihC/FbpC_Bp        VTQTSAS-YT TYADQKLYLA KGELYDLIKA IESTTTTYDA YIKSTGLSHG SKFSQVETIL KEVKGLIKQA --IK 349
CihC/FbpC_Bt        TNDTPIS--- --ANQSLNEA KSILQDLIQA VEKVSSTYDA YAKSTGIGFG SKFSEVEKNL KDAKGSIQKA KTIR 363
CihC/FbpC_Bh-FRO    SSETPQSNYV TEADKSLYLA KDKLYDLIKA VESSANTYDA YAKRTGIGHG SKFSEVENHL KDAKSLIKKA --LK 374
CihC/FbpC-N_Bh-HS1  ---------- ---------- ---------- ---------- ---------- ---------- ---------- ---- 173
CihC_Br             IAEDELYRAI -YILQDLFWS KYGLYDLVEA VGKTSSTYEA YVKRSGLGF- AKISEVEQSL KKAKELLAKR KNSK 356
CihC-N_Br           ---------- ---------- ---------- ---------- ---------- ---------- ---------- ---- 175
CihC-C_Br           IAEDELYRAI -YILQDLFWS KYGLYDLVEA VGKTSSTYEA YVKRSGLGF- AKISEVEQSL KKAKELLAKR KNSK 162
BBK32               TAANFVYIND THAKRKLENI EAEIKTLIAK IKEQSNLYEA YK-----AIV TSILLMRDSL KEVQGIIDKN GVWY 354
                                                                                          K327
```

Supplementary figure 2. Sequence alignment of C1r-binding proteins of relapsing fever borreliae and BBK32 of *Borrelia burgdorferi* B31. For sequence analyses, the CLC sequence Viewer 8.0 (QIAGEN Aarhus A/S, Denmark) and EMBOSS Needle (https://www.ebi.ac.uk) was used. The identified amino acid residues at positions 248 and 252 of CihC/FbpC ortholog of *B. hermsii* involved in binding of C1r are indicated (light orange) and arginine-248 and lysine-327 in BBK32 of *B. burgdorferi* B31 are highlighted in light green.
