# Supplementary material for: Multifunctional interaction of CihC/FbpC orthologs of relapsing fever spirochetes with host-derived proteins involved in adhesion, fibrinolysis, and complement evasion
Source: Front Immunol. 2024 Apr 25;15:1390468. doi: 10.3389/fimmu.2024.1390468 (PMC11079166; doi:10.3389/fimmu.2024.1390468)
Supplement: Supplementary file 4 [file Presentation_3.pdf]

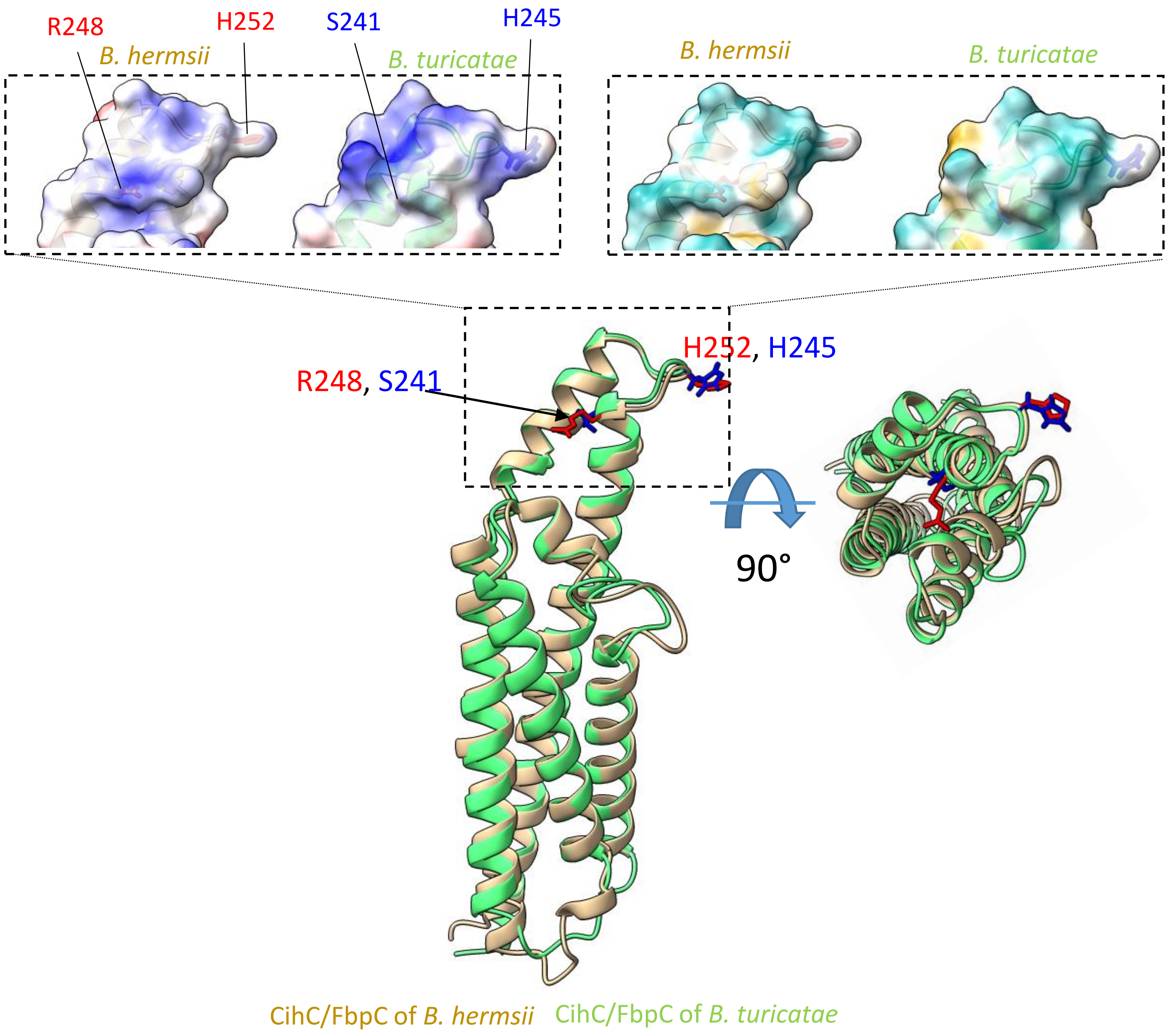

Supplementary figure 3. Superimposition of AlphaFold2-predicted structures of *B. hermsii* (brown) versus *B. turicatae* (light green) obtained using the Matchmaker function of ChimeraX. The top of the figure shows the space filling models corresponding to the electrostatic distribution (left inset; positive charges in blue, negative in red) or hydrophobic/hydrophilic residues (right inset; hydrophobic residues in orange/brown, hydrophilic residues in cyan). The residues R248 and H252 from *B. hermsii*, located in the α1 helix and the short loop connecting the α1 and α2 helices, respectively, are shown in red, while the matching residues in *B. turicatae* are shown in blue. In the bottom right part of the figure, the superimposed ribbon structure was rotated 90° towards the viewer.
